# Experience-Dependency of Reliance on Local Visual and Idiothetic Cues for Spatial Representations Created in the Absence of Distal Information

**DOI:** 10.3389/fnbeh.2017.00092

**Published:** 2017-06-06

**Authors:** Fabian Draht, Sijie Zhang, Abdelrahman Rayan, Fabian Schönfeld, Laurenz Wiskott, Denise Manahan-Vaughan

**Affiliations:** ^1^Department of Neurophysiology, Medical Faculty, Ruhr University BochumBochum, Germany; ^2^International Graduate School of Neuroscience, Ruhr University BochumBochum, Germany; ^3^Institute for Neural Computation, Ruhr University BochumBochum, Germany

**Keywords:** sensory, hippocampus, CA1, place cell, distal cue, spatial

## Abstract

Spatial encoding in the hippocampus is based on a range of different input sources. To generate spatial representations, reliable sensory cues from the external environment are integrated with idiothetic cues, derived from self-movement, that enable path integration and directional perception. In this study, we examined to what extent idiothetic cues significantly contribute to spatial representations and navigation: we recorded place cells while rodents navigated towards two visually identical chambers in 180° orientation via two different paths in darkness and in the absence of reliable auditory or olfactory cues. Our goal was to generate a conflict between local visual and direction-specific information, and then to assess which strategy was prioritized in different learning phases. We observed that, in the absence of distal cues, place fields are initially controlled by local visual cues that override idiothetic cues, but that with multiple exposures to the paradigm, spaced at intervals of days, idiothetic cues become increasingly implemented in generating an accurate spatial representation. Taken together, these data support that, in the absence of distal cues, local visual cues are prioritized in the generation of context-specific spatial representations through place cells, whereby idiothetic cues are deemed unreliable. With cumulative exposures to the environments, the animal learns to attend to subtle idiothetic cues to resolve the conflict between visual and direction-specific information.

## Introduction

Place cells in the hippocampus are an important part of the neuronal substrates for spatial navigation and spatial memory (O’Keefe and Nadel, 1978). They exhibit high-frequency discharges when animals traverse a specific location within an environment. Their firing patterns are context-specific and reproducible under identical conditions after weeks and even months (Thompson and Best, 1990). External sensory cues provide a key stream of information for place cells, and their activity is acutely controlled by both visual cues (O’Keefe and Conway, 1978; Muller and Kubie, 1987; Rotenberg and Muller, 1997) as well as olfactory cues (Save et al., 2000; Anderson and Jeffery, 2003; Zhang and Manahan-Vaughan, 2015). Place cells also retain highly defined firing patterns in the absence of salient sensory cues (Quirk et al., 1990; Save et al., 2000; Zhang and Manahan-Vaughan, 2014), which suggests that their activity is dependent on additional information derived from idiothetic cues (McNaughton et al., 1996, 2006).

A number of studies have addressed the extent to which hippocampal encoding of space, as reflected by context-dependent place cell firing, relies on distal, proximal and idiothetic cues. For example, place cell firing in visually identical environments using various multi-compartment settings was examined (Skaggs and McNaughton, 1998; Tanila, 1999; Fuhs et al., 2005; Paz-Villagrán et al., 2006; Spiers et al., 2015; Grieves et al., 2016). Tanila (Tanila, 1999) recorded hippocampal place cells mainly from the CA3 subfield, during navigation within two visually identical boxes that were illuminated from above by four incandescent lights and were connected door-to-door in the *same* orientation. He showed that distinct spatial representations occur in both boxes despite their ostensible visual similarities. Skaggs and McNaughton (Skaggs and McNaughton, 1998) recorded place cells from the CA1 region whilst rats explored two boxes that were visually similar in terms of their visual cues and directional orientation, and were connected with a lateral corridor. Recordings were conducted during illuminated conditions. Here, spatial representations in the boxes were different depending on the individual rat and even varied within the same rat across different experimental sessions. In effect, the hippocampal maps of the two environments were a mixture of orthogonal and identical maps. Fuhs and colleagues (Fuhs et al., 2005) repeated this experiment and found no evidence for place cell remapping between boxes that had the same relative orientation. When they connected the boxes door-to-door whilst rotating one box by 180°, place cell remapping was observed. This suggests that despite allowing the animals to “know” that two identical environments existed, the hippocampus encoded these environments as if they were the same places. Furthermore, cumulative exposure of the animals to the environment influenced whether place fields in same orientation environments remapped. This latter finding suggests that allowing the animal to learn and remember the environments may have facilitated attendance to more subtle cues that allowed it to better discriminate between the environments. Given that, in these two latter studies, distal (extra-environmental) cues were not available, this also suggests that the relative reliance of the animals on local visual and idiothetic cues strongly impacts on their perception and spatial representations of visually similar environments.

In the present study, we explored to what extent path integration, derived from direction-specific information, or proximal visual cues, is used as the primary basis for hippocampal encoding under circumstances where distal cues are absent. To force the animals to particularly rely on these kinds of cues, we conducted experiments in the dark, in white noise and in the absence of reliable olfactory cues. Specifically, we recorded place fields during navigation in two visually identical environments in the dark, one of which was rotated by 180° with respect to the other. Access to the environments occurred via a corridor and fluorescent cue cards were used as local visual cues. Place fields were identical in both environments, despite their 180° orientation.

When the rats were allowed direct access between the two environments (via a corridor) and had the opportunity to understand that two environments existed, place fields did not remap. Repeated exposures to the paradigm ultimately resulted in place field remapping when unrestricted “simultaneous” access to both environments was possible. These findings support that in paradigm-naïve rats, an initial reliance on local visual cues occurs (as indicated by identical place fields in the mirrored environments), even when both chambers could be accessed simultaneously by the rats. This was gradually succeeded by separate spatial representations that depend on idiothetic cues. Subsequent re-exposure of the animals to the same chamber sequence (separate exploration of identical mirrored chambers followed by dual exploration) showed that memory of past experience impacts on hippocampal processing of the re-exposure, whereby the animals predominantly rely on idiothetic as opposed to local visual cues (indicated by rapid place field remapping upon simultaneous exposure to both chambers). Exposure to the same paradigm in illuminated conditions, not surprisingly, resulted in complete reliance on distal visual cues.

Taken together, these data suggest that under conditions where the rats are naïve to the paradigm, local visual cues predominate in the generation of anchored place fields in darkness. Prior experience of learning about the spatial environment impacts upon this strategy however: re-experience of the experimental paradigm revealed that the integration of idiothetic information gradually increases and becomes the predominant cue for place fields. But if a choice can be made between relying on local visual, direction or distal cues, then distal cues are deemed the most reliable source of information.

## Materials and Methods

### Subjects

The present study was carried out in accordance with the European Communities Council Directive of September 22nd, 2010 (2010/63/EU) for care of laboratory animals. All experiments were performed according to the guidelines of the German Animal Protection Law and were approved by the North Rhine-Westphalia (NRW) State Authority (Landesamt für Naturschutz, Umweltschutz und Verbraucherschutz, NRW). All efforts were made to reduce the number of animals used.

Ten Male Long-Evans rats (8–9 weeks old) were housed individually and maintained on a 12-h light/12-h dark cycle. The animals were given sufficient food to maintain 90% of their free-feeding weight and *ad libitum* access to water. They were handled individually for 10 min per day, 1 week before surgery.

### Electrophysiological Single-Unit Recordings

One lightweight microdrive (Axona Ltd, St. Albans, UK) was chronically implanted in each rat (10–11 weeks old at the time of surgery). Each microdrive held four tetrodes made of four twisted bundles of Formvar-coated platinum-iridium wires (19 μm, California Fine Wire, Grover Beach, CA, USA). Tetrodes were inserted into a cannula attached to the microdrive and were strengthened with cyanoacrylate glue at the base of the outlet of the cannula. One full rotation of the mechanical drive produces a vertical movement of 200 μm, without rotating the cannula or the electrodes. Each rat was chronically implanted with a microdrive as described previously (Zhang and Manahan-Vaughan, 2014). Briefly, animals were deeply anesthetized with sodium pentobarbital (52 mg/kg i.p.) and placed in a stereotaxic frame. A hole (1.1 mm diameter) was drilled in the *os parietale dexter* above the hippocampal CA1 region, 3.8 mm posterior to bregma and 3.0 mm lateral to the *sutura sagittalis*. The dura mater was pierced and the tetrodes (attached to the microdrive) were lowered into the right hemisphere to a depth of 2.5 mm from the skull. Three additional small jewelers’ screws were inserted into the *os frontale*, *os parietale sinister* and *os occipitale* that served to stabilize the microdrive, which was then fixed to the rat’s had with dental cement (Paladur, Heraeus Kulzer GmbH, Hanau, Germany). An analgesic, meloxicam (0.2 mg/kg, i.p.) was given prior to surgery and both 24 h and 48 h after surgery. Animals recovered for at least 7 days from surgery before any screening started.

Screening of place cells was performed once, or twice, daily in a square chamber that was visually distinct from the test environment. Neural activities were passed through AC-coupled, unity-gain operational amplifiers, which were mounted on a headstage (Axona Ltd, St. Albans, UK) connected close to the rat’s head through a socket that fitted onto the microdrive plug. The headstage was linked to a pre-amplifier via lightweight hearing-aid wires. The buffered signal from the headstage was amplified 10,000–20,000 times in the pre-amplifier and then digitized (48 kHz) and bandpass-filtered (0.6–6 kHz) in a dacqUSB system unit (Axona Ltd, St. Albans, UK). The signal from each tetrode could be separately distinguished, being referenced to one channel of another tetrode. The acquired waveforms were identified and sorted offline using TINT software (Axona, Ltd, St. Albans, UK), which allows spike sorting based on multiple features. We used the K-mean algorithm, included in the software, to discriminate spikes based on spike duration, spike amplitude, maximum and minimum spike voltages and their time of occurrence. The resulting clusters were either combined, or isolated manually, depending on the cluster shape (Tsanov et al., 2014; van de Ven et al., 2016). After the cluster cutting, firing rate maps for each cell were visualized using TINT which divided the camera view arena into 64 × 64 square bins with a side length of 2.5 cm. The firing rate for a given cell in each *bin* indicated the spike number divided by dwelling time in that bin. The resulting matrix encodes the firing rate map and served as the basis of further analysis. The firing rate maps were smoothed and presented in color with 0 Hz in blue and the highest firing rate in red. If no appropriate cell activity was identified, the tetrodes were advanced 25–50 μm and rats were returned to their home cages for at least 2 h. The maximum movement of tetrodes was 150 μm per day. The position of the rat was monitored by a video camera mounted directly above the arena and converted into *x-y* coordinates by a tracking system (Axona Ltd, St. Albans, UK) that detected a small infra-red light mounted on the headstage near the rat’s head.

### Behavioral Protocol

**Figure 1:**
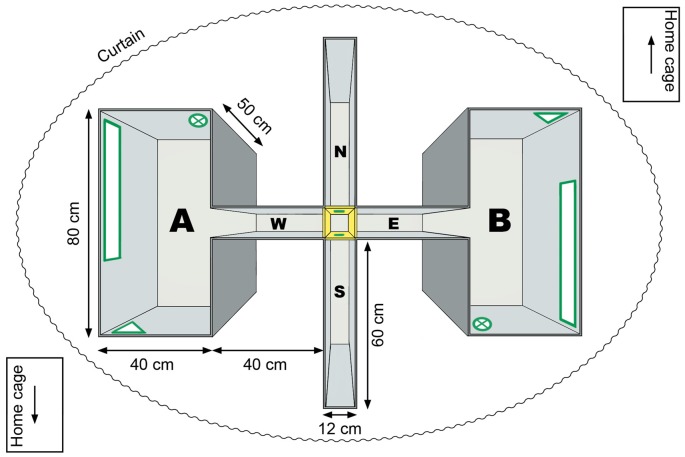
**Experimental apparatus**. Experiments were conducted in a four-arm maze, that contained two ostensibly identical chambers. The apparatus featured two entry points, on the north and south entry arms and two identical chambers (“A” and “B”), at the end of the east and west corridor. The two chambers were visually identical but flipped around the east (E)-west (W) axis of the apparatus. In the center of the maze, four doors (shown in yellow) separated the north (N)-south (S) entry arm and east-west corridor. Three cue cards (in geometrical forms) were attached to the walls inside the chamber (shown in green). An additional cue card was placed on the door at the end of the entry arm. A curtain surrounded the apparatus. Between trials rats were returned to their home cages that were surrounded by three gray plastic walls (not shown) with a cue at the top end of the cage (arrow).

Experiments were conducted in a four-arm maze with two rectangular chambers, called the twin-chamber setup; the apparatus features two entry points in the north (N) and the south (S) arm and the two rectangular chambers were connected to the east (E) and the west (W; corridor) arm (Figure 1). The chamber at the end of the west arm is referred to as “**A**” and at the end of the east arm as “**B**”. Although these did not correspond to compass orientations, the arms are assigned compass orientations here, for the sake of notational simplicity. All four arms could be blocked by a barrier, thus, forcing the rat to follow one particular route. With the exception of the distal cue experiments (illuminated condition), all experiments were conducted in darkness and in white noise, as described previously (Zhang et al., 2014).

Fluorescent cue cards were attached to the walls of the west and east chamber, and were placed in “identical” (180° rotated) positions in each chamber (Figure 1). This was done in order to create the illusion that the two chambers were identical. An additional cue card was placed at the end of the entry arm and was attached to the barrier blocking the other entry arm. The cue cards were made of fluorescent phosphorous sticky tape (tesa Anti-slip tape, tesa SE, Norderstedt, Germany) put on white plastic cards in different geometrical forms. They were in a green-yellow color that emits light at a wavelength of 500–600 nm, which is in the visual range of rats (Szél and Röhlich, 1992; Jacobs et al., 2001; Akula et al., 2003). In the past, we have shown that, in the dark, active visual exploration of fluorescent items triggers changes in excitability and promotes synaptic plasticity in the visual cortex (Tsanov and Manahan-Vaughan, 2007). Thus we are confident that the animals could perceive these local cues in darkness.

When trials were conducted in darkness, the only available visual cues were the abovementioned fluorescent cue cards, so that the chambers looked identical as the rats entered the west chamber from the door in its east wall, or the east chamber from the door in its west wall. New patterns of cue cards were used for different sessions. A curtain surrounded the setup to prevent the influence of distal cues during experiments that were conducted in darkness (Figure 1). Two auditory white noise sources were placed at the east and the west end of the maze to prevent spatial orientation on the basis of auditory cues.

**Figure 2:**
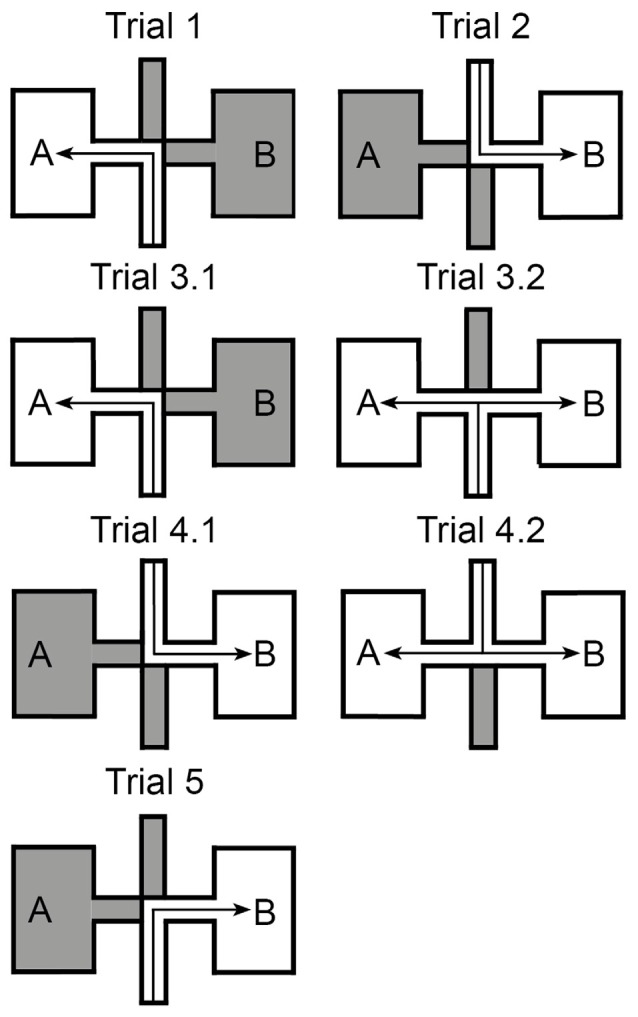
**Experimental paradigm**. The experimental paradigm consisted of multiple sessions. Each session consists of five different trials. **Trial 1**: animals were placed into the south entry arm and had to follow it until reaching the central junction, where they turned left to follow the west corridor into the chamber “A” of the maze. **Trial 2**: a mirrored repetition of trial 1, in which animals were placed into the north entry arm and reached chamber “B”. **Trial 3**: split into 3.1, in which rats performed the same task as in trial 1, and 3.2 in which the barrier that hinders free movement between the chambers is removed. Animals could thus approach both chambers by different directions. **Trial 4**: a mirrored repetition of trial 3. **Trial 5**: animals were placed into the south entry arm and turned right to reach chamber “B”.

Rats participated in one session per day comprised of five trials (Figure 2):

- **Trial 1**: animals were introduced into the maze at the end of the south (entry) arm and entered chamber “A” via the west corridor (the barriers of the north and east arm were closed).
- **Trial 2**: animals started in the north entry arm and were allowed to access chamber “B” via the east corridor. As Trial 1 and Trial 2 were rotationally equivalent, both in terms of local cues and directional self-motion cues, it was expected that in these trials the place fields in the two chambers would be highly correlated (with 180° rotation).
- **Trial 3** was split into two phases. In the first phase (Trial 3.1) rats first went from the south entry arm to chamber “A” via the west corridor (as in Trial 1). After 5 min, the barrier to the east corridor was removed (Trial 3.2) and animals were then allowed access to chamber “B” as well. Rats tended to run to and from the two chambers. The commuting of the animals, from one chamber to the other, created a conflict between the identical local visual cues in chambers “A” and “B” and idiothetic cues, which helped the rats understand that the two chambers were facing in opposite directions and were actually two separate environments.
- **Trial 4** was the same as Trial 3, except that the animals started in the north entry arm and were allowed to access the chamber “B” first (trial 4.1), followed by opening up the access route to chamber “A” (trial 4.2). This created a similar conflict as in Trial 3.
- **Trial 5**: animals started from the south entry arm and were allowed to enter the chamber “B” via the east corridor (the barriers of the north and west arm were closed).

During the five trials, animals were encouraged to move by virtue of randomly scattered tiny chocolate pieces. Rats were allowed to run freely in the restricted area in each trial. Electrophysiological recordings were performed for 5 min in Trials 1, 2 and 5. The recording time in Trials 3 and 4 was 15 min, in which 5 min were devoted to Trials 3.1 or 4.1 and 10 min were devoted to Trials 3.2 or 4.2. Recordings during the trials are indicated by 1A, 2B, 3.1A, 3.2A, 3.2B, 3A, …, 5B, whereby the number indicates the trial and the letter (A or B) indicates the chamber. For example, 3.2A indicates chamber “A” in Trial 3.2, while 3A indicates the chamber “A” in Trial 3.1 plus Trial 3.2.

During the inter-trial intervals, animals were taken out of the maze and were kept in their home cages, which were covered by an opaque cloth and surrounded on three sides by gray plastic walls. A distinctive cue was placed on the front side of the cage to ensure that the cage always had the same apparent orientation to the entry arm at the time-point of the animal’s entry to the chamber (Figure 1). The maze was carefully cleaned (to remove scents or any biological deposits that could serve as olfactory cues) and the fluorescent cue cards were “recharged” with an LED Lamp (Petzl Tikka, Petzl Distribution, Crolles, France) for approximately 15 s to rejuvenate their luminosity before each trial. Animals were disoriented before being placed into the maze by lifting and randomly rotating their covered cage with the rat inside, so that the rat did not witness that it was moved to another entry point. The disorientation protocol was similar to the one performed by Dudchenko and colleagues (Dudchenko et al., 1997) and Knierim and colleagues (Knierim et al., 1995).

Trials 1–5 were conducted as one session. After animals completed a recording session, they were housed individually for at least 6 days (with one exception where two sessions were recorded on successive days). During this time tetrodes were slightly lowered to identify new place cells. Each rat experienced up to six sessions in darkness during which place cell activity was recorded. We refer to rats that participated in their very first exposure to a session in the paradigm, as paradigm-naïve (PN), whereas experience of all subsequent sessions are referred to as paradigm-familiar (PF).

To compare results obtained in darkness with the impact of distal cues, animals participated in sessions with the lights switched on, the curtain surrounding the chambers removed, and the white noise sources absent. Under these conditions, the animals could orient themselves by using distal visual extramaze cues while exploring the seemingly identical chambers during the same five trials as in the dark. Below, sessions recorded under these conditions are termed “illuminated sessions” (IL).

### Data Analysis

Firing rate maps of place cells were analyzed with customized codes written in Matlab (MathWorks, Natick, MA, USA). To compare place fields of different trials, we calculated the spatial correlation between each of two trials. Place cells firing inside the three different sections of the paradigm, namely in: (i) the chambers; (ii) the east-west corridors; and (iii) the north-south entry arms, were analyzed separately by extracting the part of the matrix that encodes the corresponding area of the firing rate map. Here, we restrict our descriptions to results obtained from place cell firing inside the chambers, since the number of cells recorded in the east-west corridors and north-south entry arms was too low to generate statistically valid cohorts. Out of interest, we nonetheless determined that spatial correlations and the rate remapping index (Leutgeb et al., 2005) for those cells that were detected in the arms, but found no evidence of systematic effects.

When analyzing place field anchoring in the chambers, the chamber “B” was rotated by 180° before comparing it with chamber “A”.

During the course of a session, the rat entered the set-up from either the north or the south, and relative to its entry arm explored chamber “A” or “B”. This yielded four possible combinations. If we assume the chambers themselves were indistinguishable for the rat because of the local cue placement, then, depending on whether the rat relies on the local visual cues (i.e., does not distinguish chamber “A” and “B”), relies on its trajectory to distinguish chamber “A” and “B”, or uses its absolute sense of direction to distinguish the chambers, one would expect a different pattern of correlation values. If the rat mainly relies on local cues, all correlation values should be high, because the rat is not able to distinguish the chambers and should, thus, develop identical place fields in them that are anchored to the local cues. If the rat has a trajectory-based sense of direction, the correlations should only be high if the trajectory is the same, for instance between recordings obtained in 1A and 2B (left turn into the chamber in both cases). Accordingly, if the rat relies on an absolute sense of direction, high correlations can be expected between trials that involve access to a chamber using the same absolute directions, for instance during recordings in Trials 1A and 4A (which involve different approach trajectories). We refer to cells that behave according to these three cases as local cue-reliant, trajectory-reliant and “absolute” direction-reliant. We therefore consider four different groups of correlation values, namely between:

1. Recordings from two different trials with the *same* entry arm (north or south) where chambers are accessed by means of the *same* trajectory (left or right turn), which implies also the same absolute direction (chamber “A” or “B”), since the entry arm is the same,
2. Recordings from two different trials with a *different* entry arm (north vs. south) where chambers are accessed by means of the *same* trajectory (left or right turn), which implies different absolute directions (chamber “A” vs. “B”),
3. Recordings from two different trials with a *different* entry arm (north vs. south) and where chambers are accessed by means of a *different* trajectory (left turn vs. right turn), which implies the same absolute direction (chamber “A” or “B”),
4. Recordings from two different trials with the *same* entry arm (north or south) where chambers are accessed by means of a *different* trajectory (left vs. right turn), which implies also different absolute directions (chamber “A” vs. “B”).

For the first group of correlation values, there are three pairings of recordings that contribute to it, namely 1A-3A, 2B-4B, and 3B-5B, where the number indicates the trial and the letter (A or B) indicates the chamber from which the data is taken. Note that for odd numbers the entry arm was south, for even numbers it was north. The other three groups each have six pairings that contribute to them, see Table 1 for a summary of groups of correlation values and corresponding pairings, and note that the four rows (1–4) of the table correspond to the four columns of Figures 7A–C.

**Table 1:** **Summary of groups of correlation values and corresonding pairings**.

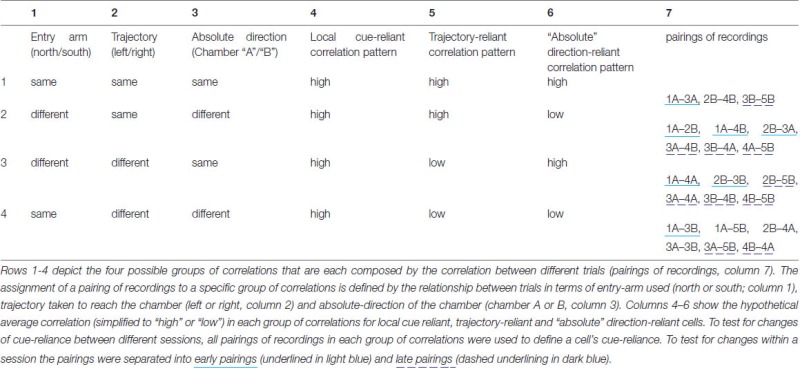

**Figure 3:**
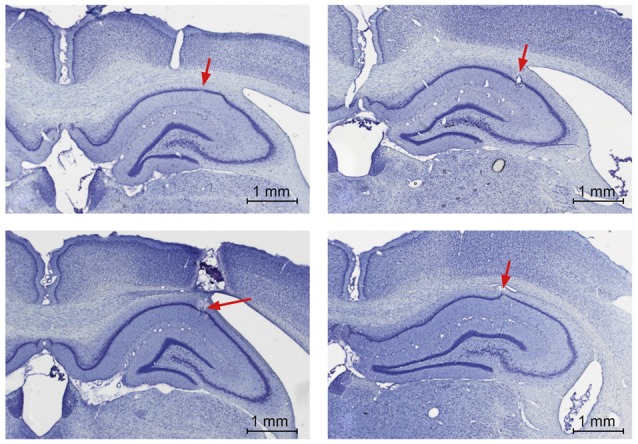
**Electrode localization in the hippocampal CA1 region**. Photomicrographs from post-mortem histological preparations conducted to verify the location of electrodes in the pyramidal cell layer of CA1. Electrode tips are indicated by the red arrows.

**Figure 4:**
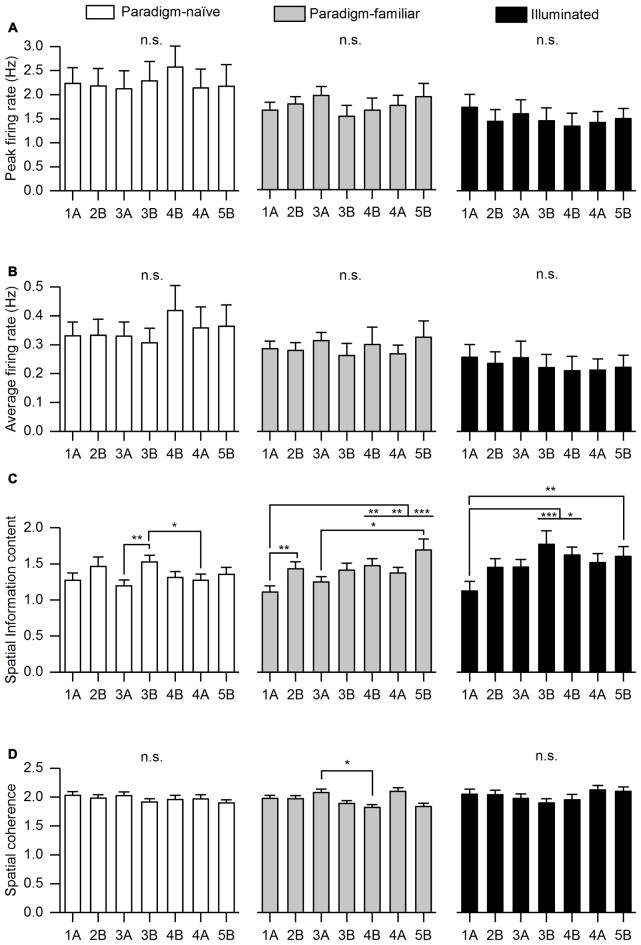
**Basic firing characteristics segregated into paradigm-naïve, paradigm-familiar and illuminated sessions**. For all conditions we analyzed: **(A)** Peak firing rate. **(B)** Average firing rate. A repeated measures analysis of variance (ANOVA) with Greenhouse-Geisser correction revealed no significant differences of peak firing rate and average firing rate in the paradigm-naïve session (*F*_(4.12, 127.707)_ = 0.411, *p* = 0.806; *F*_(2.926,90.695)_ = 0.811, *p* = 0.488), paradigm-familiar sessions (*F*_(2.56, 125.427)_ = 0.645, *p* = 0.563; *F*_(1.928,94.490)_ = 0.452, *p* = 0.63) and illuminated sessions (*F*_(1.641,41.029)_ = 0.131, *p* = 0.838; *F*_(1.312,32.808)_ = 0.139, *p* = 0.78). **(C)** Spatial information content. A repeated measures ANOVA revealed a significant difference with regard to spatial information content between trials in all conditions. In the paradigm-naive session, Bonferroni corrected pair-wise comparisons determined that spatial information content of Trial 3B was significantly higher than that of Trial 3A (*p* = 0.007) and 4A (*p* = 0.014). In the paradigm-familiar sessions, spatial information content of Trial 1A was significantly lower than that of Trial 2B (*p* = 0.004), 4B (*p* = 0.008), 4A (*p* = 0.005) and 5B (*p* = 0.001). Moreover, spatial information content of Trial 3A was significantly lower than that of Trial 5B (*p* = 0.025). In the illuminated sessions, spatial information content of Trial 1 was significantly lower than that of Trial 3B (*p* = 0.001), 4B (*p* = 0.029) and 5B (*p* = 0.009). **(D)** Spatial coherence. Pair-wise comparisons with a Bonferroni-correction followed a repeated measures ANOVA and determined that spatial coherence of Trial 3A in paradigm-familiar sessions was significantly higher than that of Trial 4B (*p* = 0.038). Asterisks signify different significance levels (*p ≤ 0.05; **p ≤ 0.01; ***p ≤ 0.001).

**Figure 5:**
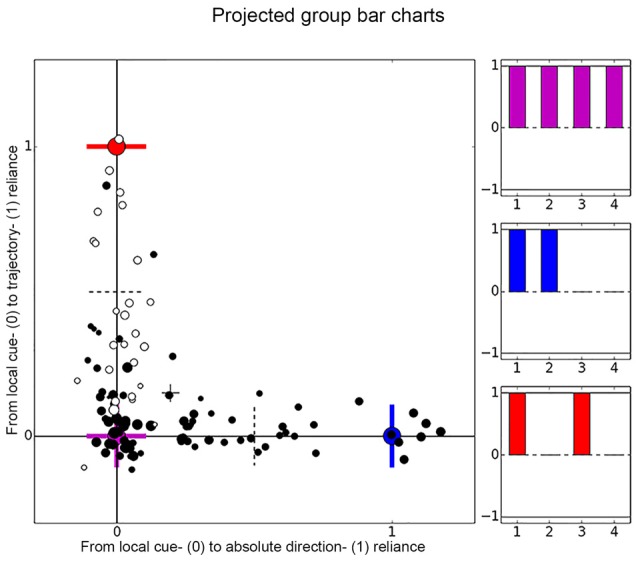
**Distribution of place cell firing patterns inside the chambers**. Each place cell is represented by a dot, black dots are used for cells recorded in darkness and white dots depict cells recorded under illuminated conditions. The larger the dot, the later the session. On the *x*-axis cells distribute between local cue-reliant (violet mark) and trajectory-reliant (blue mark) firing patterns. On the *y-axis* cells distribute between local cue-reliant and “absolute” direction- reliant (red mark) firing patterns. These endpoints correspond to idealized correlation patterns of the local cue-reliance, trajectory-reliance and “absolute” direction-reliance, that are represented by bar charts in matching colors. Each bar represents one group of correlations and relates to row 1–4 of Table 1.

**Figure 6:**
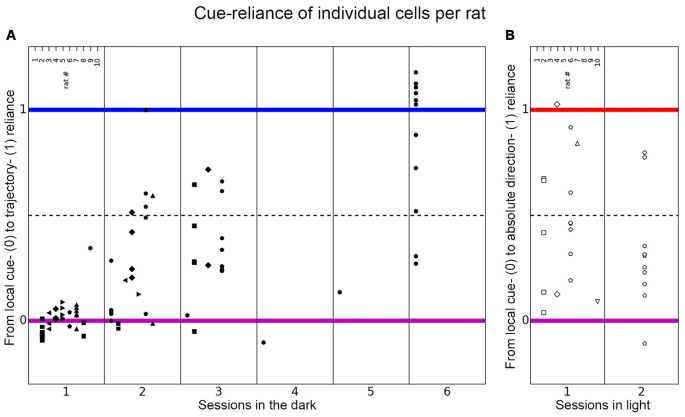
**Distribution of place cell firing patterns inside the chambers in different sessions**. The distribution of place cells of each rat is shown for each session on the axis between local cue-reliance and trajectory-reliance **(A)** and local cue-reliance and “absolute” direction-reliance **(B)**. Place cell recordings from individual rats are presented here in columns representing responses recorded in each session and are signified with different symbols. **(A)** Place cells recorded in each session in the dark distributed along the axis between local cue-reliance and trajectory-reliance. Session 1 is equivalent to the paradigm-naive session, session 2–6 correspond to the subsequent paradigm-familiar sessions. In the paradigm-naive session, cells group around the local cue-reliant endpoint, whereas in paradigm-familiar sessions they start to scatter towards the trajectory-reliant endpoint. **(B)** Place cells recorded in each session in light distributed along the axis between local cue-reliance and “absolute” direction-reliance. Cells distribute all along this axis right from the initial session that was conducted under illuminated conditions.

**Figure 7:**
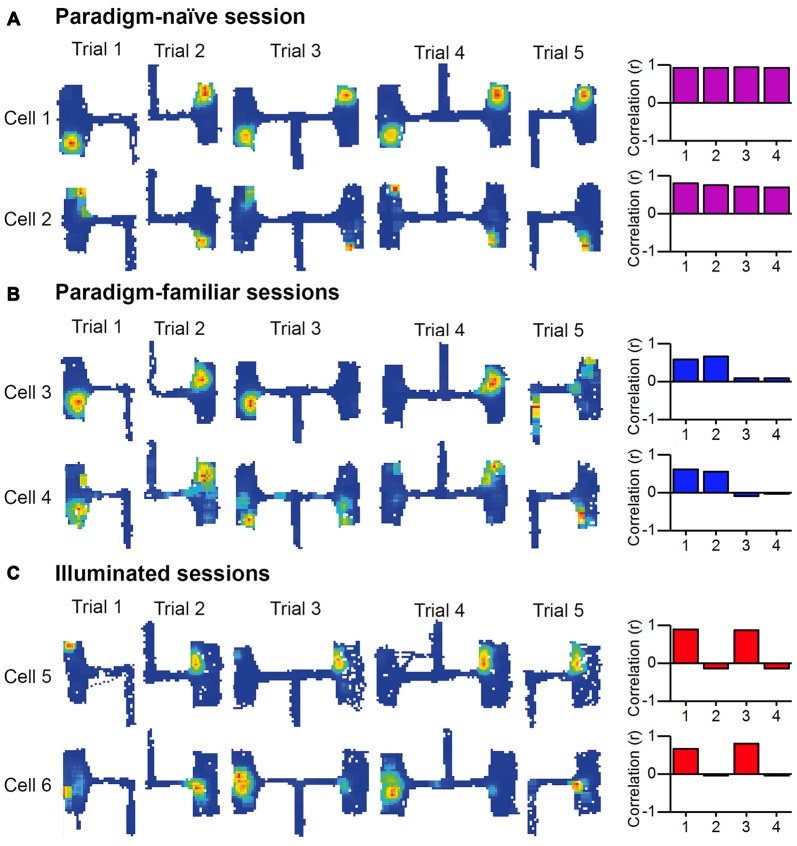
**Examples of place fields inside the chambers recorded in the paradigm-naive session, paradigm-familiar sessions and illuminated sessions**. Place fields of six place cells are shown, that were recorded during three different conditions (paradigm-naive, paradigm-familiar, illuminated). The bar charts on the right represent each cell’s correlation pattern corresponding to Table 1 based on the mean value of each group of correlations. **(A)** Paradigm-naïve session: two representative examples from two different rats that performed the experiment for the first time. Place fields are at corresponding locations in both chambers. The pattern correlates to the idealized local cue-reliant firing pattern. **(B)** Paradigm-familiar sessions: two representative examples from two different rats that performed the experiment for the second (cell 3) and third (cell 4) time. Cell 3 develops no place field inside the opposing chamber that is reached via right turn from the entry arm (3.2B, 4.2A, 5B). Cell 4 forms a new place field inside chambers of Trial 3.2B, 4.2A and 5B. The correlation pattern of both cells correlates to the idealized trajectory-reliant firing pattern. **(C)** Illuminated sessions: two representative examples of two different rats that performed the experiment under illuminated conditions. Place fields do not form at corresponding locations between both chambers, but remain at the same location within chamber “A” and “B”. The correlation pattern correlates to the idealized “absolute” direction-reliant firing pattern.

**Figure 8:**
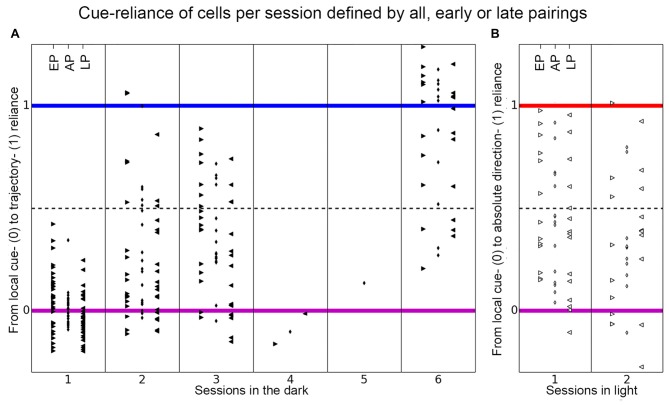
**Distribution of place cell firing patterns inside the chambers separated in early, late and all pairings**. The distribution of all recorded place cells is shown, separated into each session, on the axis between local cue-reliance and trajectory-reliance **(A)** and local cue-reliance and “absolute” direction-reliance **(B)** of place cell firing. The cue-reliance was defined by the composition of correlation values between trials in distinct groups of correlations. The small rhombus in the middle columns depicts the distribution of all place cells per session, if all pairings (AP) in each group of correlations were taken to calculate each cell’s cue-reliance. Arrowheads in the left columns that point to the right show the distribution of all place cells per session if only early pairings (EP) were used to define each cell’s cue-reliance, and arrowheads in the right columns that point to the left show the distribution if only late pairings (LP) were used (see Table 1 for a more detailed explanation). **(A)** Place cells recorded in each session in the dark distribute along the axis between local cue-reliance and trajectory-reliance of firing, separated in early, late and all pairings. There is a gradual increase of trajectory-reliance over sessions (all pairings between sessions). Within session 1 and 3 (between early and late pairings) cells became more local cue-reliant, while within session 2 and 6 no change of cue-reliance was observed. **(B)** Place cells recorded in each session in light distribute along the axis between local cue-reliance and “absolute” direction-reliance of firing, separated in early, late and all pairings. There was no change towards a preference for distal cues between sessions. Within session 1 cells became more local cue-reliant.

If [c1, c2, c3, c4] indicates the average correlation values of the four groups listed in Table 1 (rows 1–4). Then the ideal local cue-reliant cell would have the profile [c1, c2, c3, c4] = [1, 1, 1, 1], the ideal trajectory-reliant cell would have [1, 1, 0, 0], and the ideal “absolute” direction-reliant cell would have [1, 0, 1, 0] (see Table 1 columns 4–6). In order to analyze the cells in terms of their directionality, we calculated a “cue-reliance value” by projecting the four dimensional vectors [c1, c2, c3, c4] of all cells onto the two orthonormal (i.e., perpendicular and normalized) vectors [1, 1, −1−1]/2 and [1, −1, 1, −1]/2. These two select the dimensions along which cells develop from local cue-reliance [1, 1, 1, 1] to either trajectory-reliance [1, 1, 0, 0] or “absolute” direction-reliance [1, 0, 1, 0], respectively, while ignoring any change in the average over all four values. Figure 5 shows the cells in this 2D coordinate system, where the origin [0, 0] (lower left corner) indicates ideal local cue-reliant cells, [1, 0] (lower right corner) indicates ideal trajectory-reliant cells, and [0, 1] (upper left corner) indicates “idealized” “absolute direction”-reliant cells. Figures 6 and 8, show just one of the two dimensions against some other parameter, such as session and cell number. In other words, the first dimension in the plots from local cue-reliant to trajectory-reliant is (c1 + c2 − c3 − c4)/2; the second dimension in the plots from local cue-reliant to “absolute” direction-reliant is (c1 − c2 + c3 − c4)/2. These values are not normalized to the interval [0, 1], thus a cell’s cue-reliance can exceed this interval. For instance, if for a trajectory-reliant cell c1 and c2 assume the ideal value of 1 and c3 and/or c4 by chance assume a negative value, then (c1 + c2 − c3 − c4)/2 may be greater than one. Similarly, if for a local cue-reliant cell c3 and c4 assume the ideal values of 1 but c1 and c2 assume slightly smaller values, then (c1 + c2 − c3 − c4)/2 may be negative.

To allow statistical analysis and to test for changes of cue-reliance between sessions, all place cells of different rats that were recorded in a particular session were pooled (see Table 1; all pairings (AP) in Figure 8). The cue-reliance of each cell was defined by the average correlation value of all pairings in each group of correlations. To investigate changes of cue-reliance within a session we subdivided the pairings of recordings in each group of correlations in early pairings (EP) and late pairings (LP; see Table 1; Figure 8). The two dimensions ignored are [1, 1, 1, 1]/2 indicating the average correlation, which we believe largely reflects the quality of the cell and/or recording and not the cue-reliance, and [1, −1, −1, 1]/2, which we interpret as noise.

We also applied principal component analysis to verify this approach (not shown). Using it without removing the mean from the data gave very similar but less clear results if we used the second and third principal component. The first principal component was close to the average dimension [1, 1, 1, 1]/2, and the last principal component was close to the noise dimension [1, −1, −1, 1]/2 with small variance, supporting the interpretation as noise.

**Figure 9:**
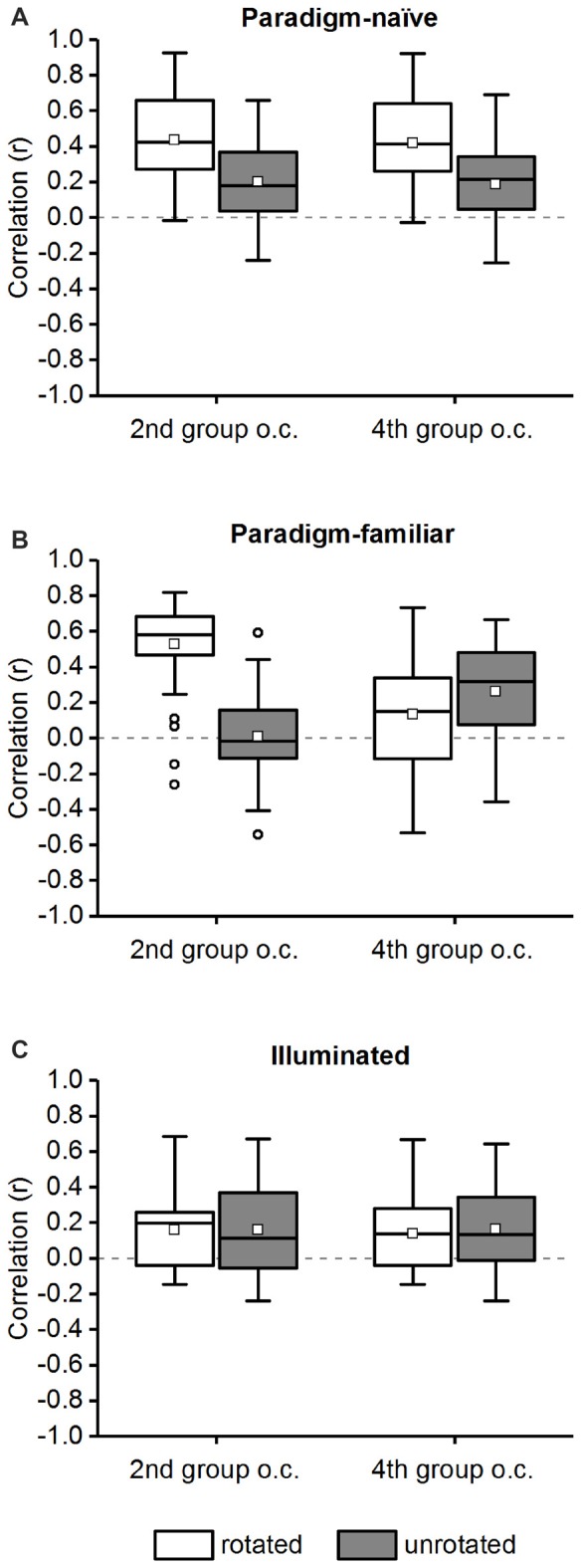
**Comparison of correlation values for the rotated and unrotated chamber “B” in the 2nd and 4th group of correlations**. The average correlation of the 2nd and 4th group of correlations (“group o.c.”; see Table 1) is shown for chamber “B” in a rotated and unrotated configuration in the paradigm-naïve session **(A)**, paradigm-familiar **(B)** and illuminated **(C)** sessions. The data are displayed in box plots: the bottom and the top of the box represent the first and the third quartiles (25%–75%). The band and the small square inside the box represent the median and mean. The upper tip of the whisker (vertical t-bar) depicts the maximum value and the lower tip reflects the minimum value, that is still within the 1.5 interquartile range (IQR). Values exceeding the 1.5 IQR are marked with circles. **(A)** In the paradigm-naïve session correlation values for the unrotated chamber “B” were significantly lower compared to those for the rotated chamber “B” in both the 2nd and 4th group of correlations. **(B)** In paradigm-familiar sessions correlation values for the unrotated chamber “B” were significantly lower compared to those for the rotated chamber “B” in the 2nd and significantly higher in the 4th group of correlations. **(C)** In illuminated sessions correlation values for the unrotated chamber “B” were not significantly different compared to those for the rotated chamber “B” neither in the 2nd nor in the 4th group of correlations.

We also calculated the correlation without rotating chamber “B” and compared the results to the rotated data (rotated vs. unrotated; Figure 9) in the 2nd and 4th group of correlations (not rotating chamber “B” has no effect on the 1st and 3rd group of correlations). If place cells were mainly influenced by local cues, correlation values would be lower in the unrotated group. If local cues have *no* influence on place cells, i.e., place cells fire in an “absolute” direction-reliant manner, no significant difference will occur between the rotated or unrotated correlation values in both groups of correlations (because both chambers are regarded as comprising two different environments and place cell firing will thus be different in both chambers independent of the trajectory used by the animal, or the local cues that are available).

Spatial information and spatial coherence were also calculated for each rate-map in individual chambers, using methods described by others (Muller and Kubie, 1989; Skaggs et al., 1992). For empty values resulting from failure of a cell to fire in a specific trial, the series mean was used for statistical analysis using an analysis of variance with repeated measures (ANOVA).

### Statistics

For statistical analysis we used the Wilcoxon rank sum test, Kruskal-Wallis rank sum test, Wilcoxon signed rank test as well as an ANOVA with repeated measures. The *p*-values were corrected for multiple comparisons using Bonferroni’s correction method. We used the *r*-value to compute the effect of size instead of Cohen’s *d* distance, because of the non-normal distribution of our data. We used R statistical environment (R Core Team (2013)). R: a language and environment for statistical computing. R Foundation for Statistical Computing, Vienna, VA, Austria) and SPSS (IBM Corp. Released 2016. IBM SPSS Statistics for Windows, Version 24.0. Armonk, NY, USA: IBM Corp) to compute the statistics. We used also coin package in our computation (Hothorn et al., 2006, 2008).

### Histological Analysis

The correct location of the recording electrodes was verified by postmortem histological analysis (Figure 3). The tissue was fixed, then coronal slices were obtained and Nissl stained (Manahan-Vaughan et al., 1998).

## Results

A total number of 128 place cells were recorded in 10 male Long Evans. The firing characteristics of the place cells were segregated according to paradigm-naïve, paradigm-familiar and illuminated sessions (Figure 4). Average firing rates and peak firing rates remained stable throughout all trials. Spatial information content and spatial coherence differed between trials in some cases (Figure 4).

Table 2 shows the distribution of place cells over conditions (paradigm-naïve, paradigm-familiar, or illuminated) and sections of the setup (chambers, corridors, entry arms). Some place cells fired in more than one section. Here, we present results obtained from the 108 place cells that fired inside the chambers.

**Table 2:** **The table shows the number of animals, cells and place fields used in the study**.

Condition	# Rats	# Cells	# Chamber place fields	# Corridor place fields	# Entry arm place fields
Paradigm-naïve session	8	36	32	11	12
Paradigm-familiar sessions	7	67	50	17	20
Illuminated sessions	5	28	26	6	7
Total	10	128	108	34	39

*Place fields were analyzed separately in the chambers, east-west corridor arms and north-south entry arms (data from corridors and entry arms is not shown). All sessions were sorted into three conditions. Most rats went through Trials 1–5 in multiple sessions. We distinguished paradigm-naïve and paradigm-familiar sessions, both in darkness and in the illuminated sessions*.

### Distribution of Place Cell Firing Patterns Inside the Chambers

Based on each cell’s correlations values, we projected the data onto a two-dimensional coordinate system and distributed all cells according to their correlation pattern along the two axes (Figure 5). There are three highlighted end-points representing idealized correlation patterns for local cue-reliance, trajectory-reliance and “absolute” direction-reliance of firing patterns. “Idealized” means, that we defined a hypothetically ideal correlation pattern for each sense of direction. Contrary to expectations, cells did not form clusters around the idealized end-points. Rather they scattered along the *x*- and *y-axis* between local cue-reliance and trajectory reliance, and between local cue-reliance and “absolute” direction reliance. Cells were not placed between “absolute” direction-reliance and trajectory-reliance indicating that these two senses of direction were mutually exclusive. Cells that exceeded the idealized endpoints, e.g., those six cells on the *x*-axis, showed negative correlation values where a correlation value of zero was defined as ideal. However, there we found no evidence that negative correlations occurred frequently.

### Place Cells Recorded in the Paradigm-naïve Session Are Mainly Local Cue-Reliant

In the paradigm-naïve session, all the rats’ cells, except for one cell recorded in rat 9, were positioned around the idealized local cue-reliant endpoint (median cue-reliance value = 0.0076 on the axis between local cue-reliance and trajectory reliance; Figures 6A, 8A all pairings), which implies that cells mainly behaved in a local cue-reliant manner.

Two representative cells of the paradigm-naïve session are shown in Figure 7A, together with their respective bar chart. The place fields are at corresponding locations in the two chambers (i.e., the locations in chamber “B” are rotated 180° compared to those in chamber “A”), indicating that the rat does not distinguish the two chambers. This behavior was maintained even in Trials 3 and 4 where the barrier between the east and west corridor was removed and the rat could commute from one chamber directly to the other. Thus, under these circumstances place field anchoring was dominated by local visual cues.

### In Paradigm-Familiar Sessions, Place Cells Gradually Shift Towards Trajectory-Reliance

From session 2 onwards, place fields no longer grouped around the local cue-reliant endpoint (median cue-reliance for session 2 = 0.1973, session 3 = 0.2757 and session 6 = 1.0241 on the axis between local cue-reliance and trajectory reliance; Figures 6A, 8A all pairings). Rather, they started to scatter along the axis between local cue-reliance and trajectory-reliance, with more cells positioning more closely towards the idealized trajectory-reliant endpoint.

Figure 7B shows recordings from two representative cells and their respective bar charts recorded during session 2 (cell 3) and 3 (cell 4). In Trials 1A, 2B, 3A, 4B (all involving a left turn into the chamber) the place field locations remained comparable. But in contrast to place fields that occurred in the paradigm-naïve session, we now observed that in Trials 3B, 4A, and 5B place fields mapped at different locations that were coherent with each other. This indicates that the rat had begun to distinguish two chambers based on its trajectory and presumably because it began to learn and remember its previous paradigm experiences.

This occurred in a gradual process, rather than a discrete transition. Furthermore, place cells within one rat and session did not necessarily behave coherently, as place fields sometimes scattered quite a lot along the axis for local cue-reliance and trajectory-reliance.

### Learning to Disambiguate the Two Chambers in Darkness Based on Idiothetic Cues Requires an Inter-Session Interval. In the Course of a Session, Place Cells Become More Local Cue-Reliant

All place fields detected within a session in the dark were grouped together across all rats (Figure 8A). Within a session, we distinguished early pairings (EP, left) and late pairings (LP, right; see Table 1), we also obtained values for all pairings (AP, middle). Not considering sessions 4 and 5, which have just one cell, late pairings have on average lower values than early pairings, i.e., the cells became more local-cue reliant within a session. Across sessions, however, cells demonstrated an increasing trajectory-reliance, as can be seen from the values for all pairings, which increase on average from session to session.

The increase in trajectory-reliance was not something that developed within an individual session, rather it occurred *across* sessions. The increase in trajectory-reliance of place cells across sessions was confirmed by a Kruskal-Wallis chi-squared test (*χ*^2^ = 47, *df* = 5, *p*-value < 10^−9^). Wilcoxon rank sum test revealed a highly significant shift towards trajectory-reliance from session 1 (median = 0.0076) to session 2 (median = 0.1973; *w* = 116, *p*-value = 0.0008, *r* = 0.5128), from session 1 (median = 0.0076) to session 3 (median = 0.2757; *w* = 46, *p*-value < 10^−5^, *r* = 0.6781), from session 1 (median = 0.0076) to session 6 (median = 1.0241; *w* = 2, *p*-value < 10^−8^, *r* = 0.7385), from session 2 (median = 0.1973) to session 6 (median = 1.0241; *w* = 16, *p*-value = 0.0001, *r* = 0.6953) and from session 3 (median = 0.2757) to session 6 (median = 1.0241; *w* = 20, *p*-value = 0.0014, *r* = 0.6534). However, there was no significant increase in trajectory-reliance between session 2 (median = 0.1973) and session 3 (median = 0.2757; *w* = 110, *p*-value = 0.63, *r* = 0.2719; Figure 8A, all pairings).

When each session was tested individually by comparing early pairings and late pairings, we found that there was no shift towards trajectory-reliance. We even observed a significant shift towards a local cue-reliant firing pattern within session 1 (median_EP_ = 0.0634 and median_LP_ = −0.0462; Wilcoxon signed-rank test: *v* = 357, *p*-value = 0.03211, *r* = 0.267) and session 3 (median_EP_ = 0.4534, median_LP_ = 0.1231; Wilcoxon signed-rank test: *v* = 126, *p*-value = 0.01743, *r* = 0.4018). For session 2 and 6, there was no shift neither towards trajectory-reliance nor local cue-reliance (session 2: median_EP_ = 0.1166, median_LP_ = 0.1104; Wilcoxon signed-rank test: *v* = 110, *p*-value = 0.3038, *r* = 0.1687; session 6: median_EP_ = 1.0180, median_LP_ = 0.8658; Wilcoxon signed-rank test: *v* = 46, *p*-value = 0.2783, *r* = 0.2464; Figure 8A, EP and LP).

Learning to disambiguate the two chambers using idiothetic cues therefore did not happen *within* sessions but rather developed in the interim *between* sessions. This suggests that experience, consolidation and cumulative learning about the paradigm experience was taking place.

### Place Cells Recorded in Illuminated Sessions Are Strongly Influenced by the Presence of Distal Cues

Place cells recorded in illuminated sessions were widely distributed along the axis between local cue-reliance and “absolute” direction-reliance (Figures 6B, 8B). Between sessions there was no significant shift towards local cue-reliance or “absolute” direction-reliance (Session 1: median cue-reliance = 0.4462; session 2: median cue-reliance = 0.2809 on the axis between local-cue reliance and “absolute” direction-reliance; Wilcoxon rank sum test: *w* = 102, *p*-value = 0.26, *r* = 0.2273; Figure 8B all pairings). Within session 1 there was a significant reduction of “absolute” direction-reliance between early pairings (median = 0.4316) and late pairings (median = 0.3801; Wilcoxon signed-rank test: *w* = 77, *p*-value = 0.026, *r* = 0.38915), but not in session 2 (median_EP_ = 0.1057, median_LP_ = 0.3895; Wilcoxon signed-rank test: *v* = 21, *p*-value = 0.55, *r* = 0.1481; Figure 6B, early pairings and late pairings).

Figure 7C shows recordings from two representative cells and their respective bar chart under illuminated conditions. Even in Trial 2, before being able to run from one chamber directly to the other one, place fields in the two different chambers were not at corresponding locations, indicating that the rat was able to distinguish the east and west chambers despite identical visual local cues, suggesting that distal cues were the dominant cue for spatial encoding under these circumstances.

### Comparison of Correlation Values with Rotated and Unrotated Chamber “B”

As mentioned above, we defined four possible groups of correlations (Table 1). In the 2nd (same trajectory, different chamber) and 4th (different trajectory, different chamber) group of correlations, chamber “B” was rotated by 180° before comparing it with chamber “A”. We also calculated the correlation without rotating chamber “B” and compared these values with the ones obtained from the rotated comparisons (Figure 9, rotated vs. unrotated). For paradigm-naïve sessions the unrotated comparisons showed a significantly reduced average correlation in the 2nd (median_rotated_ = 0.422, median_unrotated_ = 0.1775; Wilcoxon rank sum test: *w* = 778, *p*-value = 0.0002, *r* = 0.4464) and the 4th (median_rotated_ = 0.4144, median_unrotated_ = 0.2128; Wilcoxon rank sum test: *w* = 756, *p*-value < 10^−12^, *r* = 0.5791) group of correlations (Figure 9A). This confirms that place cells in the paradigm-naïve session were mainly local cue-reliant. In the paradigm-familiar condition there was a significantly reduced average correlation in the unrotated comparisons in the 2nd group of correlations (median_rotated_ = 0.5783, median_unrotated_ = −0.0172; Wilcoxon rank sum test: *w* = 2324, *p*-value = 0.0002, *r* = 0.7404). In the 4th group of correlations the average correlation in the unrotated comparisons was even significantly higher compared to the rotated group (median_rotated_ = 0.1511, median_unrotated_ = 0.3168; Wilcoxon rank sum test: *w* = 944, *p*-value = 0.03, *r* = 0.2109; Figure 9B). This indicates that in the paradigm-familiar sessions, local cues influenced place cells inside the chamber that was firstly visited, but were disregarded in the chamber that was reached through a different path. In illuminated sessions, there was no difference between the average correlation of rotated and unrotated comparisons neither in the 2nd (median_rotated_ = 0.1969, median_unrotated_ = 0.1134; Wilcoxon rank sum test: *w* = 347, *p*-value = 0.87, *r* = 0.0228) nor in the 4th (median_rotated_ = 0.136, median_unrotated_ = 0.1337; Wilcoxon rank sum test: *w* = 334, *p*-value = 0.94, *r* = 0.0101) group of correlations (Figure 9C), proving that cells in the illuminated sessions were mainly influenced by distal cues.

### Learning to Differentiate between Chambers Does Not Compromise Stability of Place Fields in the Chamber that Was Encountered First, Although Stability Varies Across Cells

**Figure 10:**
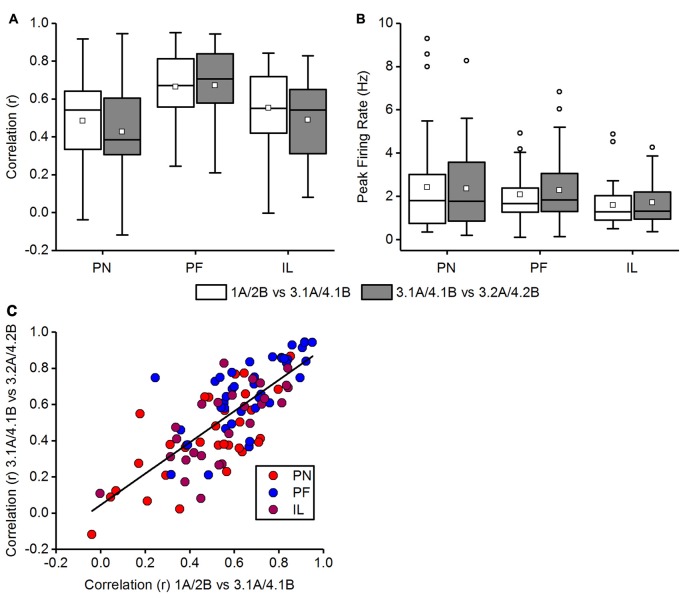
**Comparison of place fields inside the chambers before and across opening of the barrier**. In Trials 3 and 4, animals first explored a chamber after turning left (trial 3.1A and 4.1B) and established place fields in this chamber. In Trials 3.2 and 4.2, animals could explore both the initial chamber and the twin chamber. The data in **(A,B)** are displayed in box plots: the bottom and the top of the box represent the first and the third quartiles (25%–75%). The band and the small square inside the box represent the median and mean. The upper fence of the whiskers depicts the maximum value and the lower fence the minimum value that is still within the 1.5 IQR. Values exceeding the 1.5 IQR are marked with circles. **(A)** Comparison of the averaged correlation values before (1A vs. 3.1A together with 2B and 4.2B) and across (3.1A vs. 3.2A together with 4.1B vs. 4.2B) opening the barrier for the paradigm-naïve (PN) session, paradigm-familiar (PF) sessions and illuminated (IL) sessions. Correlation values do not differ significantly between before and across opening the barrier in any condition. **(B)** Comparison of the averaged peak firing rate before (1A vs. 3.1A together with 2B and 4.2B) and across (3.1A vs. 3.2A together with 4.1B vs. 4.2B) opening the barrier for the paradigm-naïve session, paradigm-familiar sessions and illuminated sessions. Peak firing rates do not differ significantly between before and across opening the barrier. **(C)** Correlation values of individual cells before opening the barrier (1A vs. 3.1A together with 2B and 4.2B) are plotted against their correlations across opening the barrier (3.1A vs. 3.2A together with 4.1B vs. 4.2B). Cells of different conditions are marked in distinct colors. A best-fit line represents the linear relationship between correlation values before and across opening the barrier.

We explored whether place fields inside the chamber that was accessed by a left turn, and experienced first, were affected by the opening of a new path that leads to the other chamber. We therefore checked the stability of place fields in chamber “A” and “B”, before the removal of the barrier had occured, i.e., between Trials 1A and 3.1A and likewise between Trials 2B and 4.1B, and then also assessed place field stability following the removal of the barrier, i.e., between Trial 3.1A and Trial 3.2A and likewise between Trial 4.1B and 4.2B. Both correlation values in each category (before and across opening the barrier) were averaged to obtain one correlation value for each category per cell. Figure 10A shows these comparisons for the paradigm-naïve session, paradigm-familiar sessions and illuminated sessions. A Wilcoxon rank sum test was used to assess spatial correlations before and across opening the barrier and revealed no significant difference in the paradigm-naïve session (median correlation of 1A/2B vs. 3.1A/4.1B = 0.54, median correlation of 3.1A/4.1B vs. 3.2A/4.2B = 0.38; *w* = 592, *p*-value = 0.28, *r* = 0.1342) and paradigm-familiar sessions (median correlation of 1A/2B vs. 3.1A/4.1B = 0.67, median correlation of 3.1A/4.1B vs. 3.2A/4.2B = 0.70; *w* = 675, *p*-value = 0.63, *r* = 0.056). For paradigm-familiar sessions this is particularly interesting, as cells begin to show an increasing trajectory-reliance and indicates that learning to distinguish the right from the left chamber does not compromise the stability of place fields in the left chamber. In illuminated sessions, the spatial correlation before and across opening the barrier was also not significantly different (median correlation of 1A/2B vs. 3.1A/4.1B = 0.549, median correlation of 3.1A/4.1B vs. 3.2A/4.2B = 0.542; *w* = 396, *p*-value = 0.29, *r* = 0.1472).

We additionally observed differences in the peak firing rate (Figure 10B), but found no significant difference between before and across opening the barrier for the paradigm-naïve session (median peak firing of 1A/2B vs. 3.1A/4.1B = 1.81 Hz, median peak firing of 3.1A/4.1B vs. 3.2A/4.2B = 1.77 Hz; *w* = 499, *p*-value = 0.86, *r* = 0.0218), paradigm-familiar sessions (median peak firing of 1A/2B vs. 3.1A/4.1B = 1.66 Hz, median peak firing of 3.1A/4.1B vs. 3.2A/4.2B = 1.84 Hz; *w* = 672.5, *p-value = 0.61*, *r* = 0.0589) or illuminated sessions (median peak firing of 1A/2B vs. 3.1A/4.1B = 1.27 Hz, median peak firing of 3.1L/4.1L vs. 3.2L/4.2L = 1.32 Hz; *w* = 314.5, *p*-value = 0.67, *r* = 0.0596).

Figure 10C shows a scatter plot of the spatial correlations across opening the barrier (Trial 3.1A vs. 3.2A and Trial 4.1B vs. 4.2B) against the spatial correlations before opening the barrier (Trial 1A vs. 3.1A and Trial 2B vs. 4.1B). The plot shows that there is a linear relationship between correlation values before and across opening the barrier. Most cells distribute along the best-fit line (*y*_*i*_ = *β*_0_ + *β*_1_ · *x*_i_ with *β*_0_ = 0.0454 (±0.04549) and *β*_1_ = 0.86304 (±0.07414); *Pearson’s r* = 0.7684) while few cells showed diverging correlation values between before and across opening of the barrier. It also reveals that some cells were relatively unstable in terms of very low correlation values in both categories. In general however, spatial correlations across opening the barrier were not systematically lower than before opening the barrier.

## Discussion

The goal of this study was to investigate the interaction between proximal and idiothetic cues in controlling place cell activity. We recorded from place cells while rats navigated through ostensibly identical environments in darkness, in the absence of distal cues. Sensory cues from auditory or olfactory sources were suppressed. Rather than have the two identical aligned environments side by side in the same compass orientation, the environments were rotated by 180° respective to one another and were accessed by means of an L-shaped corridor, thereby giving the animals the possibility of using their sense of direction to discriminate between the chambers. Initially visual cues provided the primary reliable spatial reference source, because both chambers were approached via a left turn in the access corridor, and the chambers had identical visual cues placed in mirrored positions on the chamber walls. We observed that under these circumstances the animals rely very heavily on the local visual cues in creating a spatial representation of the environment. When a corridor barrier was removed and the animal could access both chambers by commuting between the environments, using their trajectory to help them discriminate the two environments, place fields did not remap, indicating that idiothetic cues were subordinate to the local visual cues. Multiple re-exposures to the paradigm at intervals of days were necessary before the animals engaged in place field remapping in the two environments. This suggests that through cumulative learning experiences in the environments the animals learn to attend to more subtle idiothetic cues that help them use their directional sense to discriminate between the two environments. When idiothetic cues are available, the animals preferentially use these to create a spatial map of the environments. These data add to previous reports (discussed below) that show that a hierarchy exists in the implementation of distal and proximal visual cues for spatial representations, and demonstrate that in the absence of distal cues, proximal cues are used in preference to idiothetic cues.

### In Paradigm-naïve Animals, Idiothetic Cues do Not Enable Discrimination between the Two Chambers

Several studies have demonstrated that distal visual cues are a very important sensory information source for accurate spatial navigation (Shapiro et al., 1997; Save and Poucet, 2000; Parron et al., 2004). Although a combination of proximal and distal cues enables the most accurate representation, distal cues will be used in preference to proximal cues in a conflict situation (Shapiro et al., 1997). This is not an intransigent hierarchy, however: experience in an environment also impacts strongly on the reliance of a spatial map on proximal or distal cues (Renaudineau et al., 2007).

Local or distal visual cues are not the only sensory source used for spatial representations. Other sensory modalities such as olfaction are integrated into a spatial map, if they are deemed reliable enough (Save et al., 2000; Anderson and Jeffery, 2003; Zhang and Manahan-Vaughan, 2015). In addition, idiothetic information derived from the animal’s perception of its own physical movement in space comprises another important source of information (Etienne and Jeffery, 2004). Referred to as path integration, this process of integrating idiothetic information into representations based on proximal and distal cue perception, adds metric information to the spatial map that can serve to increase its precision. For path integration information to remain accurate, reference information that typically takes the form of distal cues, must be available, otherwise errors accumulate in the animal’s understanding of where it is actually located in space (Gothard et al., 1996; Redish et al., 2000; McNaughton et al., 2006; Zhang et al., 2014). The error-vulnerability of path integration-based information suggests that in a cue-preference base hierarchy, idiothetic cues may be the least preferred in a conflict situation. The data of the present study indicate that this is indeed the case. We taught our rats that the fluorescent local cue, present on the walls of the two identical chambers, comprised the only reliable source of spatial information in the environments, and used this information to convince them that only one chamber existed. Although the animals had to use a specific trajectory to access the chambers, having them do this in darkness, in the absence of other local sensory cues, resulted in place fields that were located in the same relative positions in both chambers. When we removed the corridor barrier so that the animals could freely commute between the chambers, their place fields* initially* failed to remap, suggesting that the animals continued to rely on the local visual cues, even though idiothetic information was now available.

Other studies have examined to what extent place field remap under conditions where animals navigate in ostensibly identical environments. The three studies that were described in the introduction (Skaggs and McNaughton, 1998; Tanila, 1999; Fuhs et al., 2005) are particularly relevant given the similarity of aspects of their behavioral paradigms. Strikingly, our results align with none of the outcomes of these studies. Tanila (Tanila, 1999) examined place cell firing in two identical same-orientation environments and observed place field remapping when the animals explored both environments, but here, the animals could see distal cues. Skaggs and McNaughton (Skaggs and McNaughton, 1998) also used a same-orientation environment, but they suppressed access to distal cues. They reported that place field remapping was rat-based i.e., certain rats showed remapping but this behavior also varied depending on the navigation trial. Fuhs and colleagues (Fuhs et al., 2005) reported that no remapping occurred in same-orientation environments, but remapping occurred if the environments were rotated by 180° respective to one another. Here, multiple exposures to the 180° rotated environment were sometimes needed. We observed a failure of place fields to remap in the 180° rotated environments. It is important to note that all of the abovementioned studies were conducted in the presence of a light source and that, as far as we are aware, extraneous sensory information that could have served as distal cues (e.g., noise from outside the test environment) were not suppressed. Furthermore, in contrast to our paradigm, the chambers were aligned side-by-side and access between was always via an alley that ran across one end of the aligned chambers. In our study, we deliberately suppressed access to non-visual distal cues by conducting the experiment in auditory white noise and by surrounding the environment in a thick curtain. We also cleaned the environment between trials to ensure that no useful olfactory cues were present. Each environment was reached via a separate L-shaped corridor that also were positioned in a 180° orientation. Thus, we controlled very tightly that the animals could really only depend on the local visual cue for its initial mapping of the two environments. Upon opening up simultaneous access to the two chambers, the only additional reference information the animals could use was the longer distance they had to travel to reach e.g., chamber “A” from chamber “B” and the fact that the trajectories used to access the chambers had changed. By creating these highly controlled conditions, we believe our data resolve the conflicting results of the abovementioned studies and show that in the absence of external reference information (and strong familiarity with the environments) idiothetic cues are clearly subordinate to local cues used for the generation of a spatial representation.

### In Paradigm-Familiar Animals, Idiothetic Cues Are used to Distinguish the Two Chambers

We observed that as the animals became more familiar with the chambers they learned to disambiguate the two rooms based on idiothetic cues, as reflected by the emergence of different spatial representations inside the 180°-rotated environments. Specifically, after multiple exposures to the paradigm a transition from local cue-based mapping through trajectory-based, through “absolute” direction-based mapping occurred. Trajectory-based mapping became first evident after two sessions. The experience-dependent transition from neglect of idiothetic information through to implementation of this information to enable differentiation of the environments seen in the present study, suggests that not only do the animals cumulatively learn from their past experiences in the environment, they also learn to attend very carefully to the idiothetic information that is at their disposal during free movement between the two chambers. Other studies have reported that place demonstrate a slow development of place cell remapping in morphed environments (O’Keefe and Burgess, 1996) that has been proposed to relate to long-term plasticity processes (Barry and Burgess, 2007; Lever et al., 2009). Forms of hippocampal synaptic plasticity in the CA1 region (from which we recorded our place cells) that persist for very long periods of time depend on activation of glutamatergic N-methyl-D-aspartate receptors (NMDAR; Manahan-Vaughan, 1997; Volianskis et al., 2015) and metabotropic glutamate (mGlu) receptors, such as mGlu5 (Mukherjee and Manahan-Vaughan, 2013). Interestingly, place field stabilization requires NMDAR activation and place field maintenance requires mGlu5 activation (Zhang and Manahan-Vaughan, 2014).

Our findings with regard to the effects of cumulative learning on the preferred use of local and/or idiothetic cues contrasts with postulates that an internal cognitive map is initially based on path integration, while sensory cues become associated to distinct locations (McNaughton et al., 2006). This postulate is supported, for example, by a study that examined head direction cell firing in a circular arena where the walls and floor could be rotated separately (Knierim et al., 1998). Here, after rotating the arena walls rapidly by 180°, head direction cells didn’t immediately follow the arena’s cues, but drifted towards them within a delay of about 60 s. In this study, the cue conflict was introduced by moving the rat or its surroundings, passively. In our study, the rat actively engaged in foraging and exploration of the chambers, and had no obvious frame of reference (in the form of external cues) that could be used to easily calibrate idiothetic cues. Whereas paradigm-naïve animals may have initially utilized local geometry and the local visual cues to form and stabilize their spatial map of the environment, over time, as they became familiar with the paradigm, they may have learned to attend more carefully to the idiothetic information that was available to them. By this means, accumulated self-motion information, derived from moving along the corridor between the chambers allowed the generation of a global map of the entire environment, thereby supporting the formation of new and distinct representations for each of the chambers. This interpretation is based on reports of grid cell firing in connected environments (Carpenter et al., 2015).

### In Illuminated Sessions, Rats Disambiguate the Chambers Based on Distal Visual Landmarks

When animals performed the same experiment while having access to salient distal cues, place cells firing patterns indicated that the animals rapidly and reliably differentiated between the two environments. This is not surprising: distal visual cues are known to be potent drivers of place cell activity (O’Keefe and Conway, 1978; Park and Lee, 2016) and other studies have reported that in a conflict situation they will be used in preference to local visual cues (Shapiro et al., 1997). However, the use of distal and local cues for spatial encoding is flexible and experience-dependent (Knierim, 2002; Renaudineau et al., 2007). Furthermore, the salience of visual cues determines their implementation in a spatial representation (Etienne et al., 1990, 1995). Here, we show that the same is true with regard to idiothetic cues: with cumulative learning the animal learned to increasingly rely upon idiothetic information, even though it was navigating space in complete darkness. Taken together, however, our data suggest that in a tightly controlled cue-conflict situation, the hierarchy is such that distal cues are relied on more than local cues, that are turn relied on more than idiothetic cues.

## Conclusion

In this study, we exposed rats to two ostensibly identical chambers that were positioned at 180° angles to each other and were connected by a corridor. The chambers could be entered individually (via an L-shaped corridor) from the north or the south, thereby allowing two different trajectories. Alternatively, following removal of a barrier, animals could commute between chambers by means of a straight corridor. Fluorescent cue cards served as local visual cues and animals navigated the environments in complete darkness in the absence of reliable non-visual cues. Initially the animals entered the chambers separately by means of a left turn from the entry arm, creating the illusion that only one chamber existed. They then were allowed to commute freely between the two chambers to revise their spatial representations. We recorded place cells of paradigm-naïve and paradigm-familiar rats first in darkness and then under illuminated conditions. We found that:

1. Paradigm-naïve rats develop identical place fields in the two chambers, that persist even after they can commute between chambers. This indicates that initially local visual cues dominate over idiothetic cues.
2. Paradigm-familiar rats exhibit a memory of their past experience: multiple re-exposures to the trial sessions (at intervals of days) revealed that animals become increasingly effective at disambiguating the right from the left chamber based on idiothetic cues. A transition from exclusive use of local visual cues, through integration of trajectory-based information, through reliance on idiothetic information becomes evident across sessions. This is a gradual process that requires multiple exposures to the paradigm, indicating that cumulative learning, rather than a paradigm understanding, underlies this process.
3. Under illuminated conditions, rats rely heavily on distal visual cues. This confirms previous findings, by others, that in cue-conflict situations distal landmarks are the preferred cue source.

The findings of the present study suggest that under conditions where reliable distal cues are absent and a cue-conflict is present, local visual cues are preferred above idiothetic information for the generation of a spatial representation. This is an experience-dependent process, however: cumulative learning in the cue-conflicted environment supports a gradual transition away from the dependence on local cues and towards reliance on idiothetic cues, so that ultimately this becomes the primary source of reference information used to disambiguate the identical environments.

## Author Contributions

LW and DM-V created the concept and strategy of the study. The double-box paradigm was designed by FS and LW, and was built in the lab of DM-V. Place cell recordings were conducted by FD and SZ. The data analysis approach was developed by LW and FS and implemented by FD, AR and SZ. The data were interpreted by all authors. DM-V and LW wrote the article, with contributions from all other co-authors.

## Conflict of Interest Statement

The authors declare that the research was conducted in the absence of any commercial or financial relationships that could be construed as a potential conflict of interest.
